# Mutations in the Arabidopsis homoserine kinase gene *DMR1* confer enhanced resistance to *Fusarium culmorum* and *F. graminearum*

**DOI:** 10.1186/s12870-014-0317-0

**Published:** 2014-11-29

**Authors:** Helen C Brewer, Nathaniel D Hawkins, Kim E Hammond-Kosack

**Affiliations:** Department of Plant Biology and Crop Science, Rothamsted Research, Harpenden, AL5 2JQ UK

**Keywords:** Arabidopsis, *Fusarium culmorum*, *Fusarium graminearum*, Homoserine kinase, Disease resistance, Gain of function, Fusarium head scab

## Abstract

**Background:**

Mutation of Arabidopsis *DMR1*, encoding homoserine kinase, leads to elevation in homoserine and foliar resistance to the biotrophic pathogens *Hyaloperonospora arabidopsidis* and *Oidium neolycopersici* through activation of an unidentified defence mechanism. This study investigates the effect of mutation of *dmr1* on resistance to the ascomycete pathogens *Fusarium graminearum* and *F. culmorum*, which cause Fusarium Ear Blight (FEB) disease on small grain cereals.

**Results:**

We initially found that the *dmr1*-*2* mutant allele confers increased resistance to *F. culmorum* and *F. graminearum* silique infection, and decreased colonisation of rosette leaves. Meanwhile the *dmr1*-*1* allele supports less rosette leaf colonisation but has wild type silique resistance. Three additional *dmr1* alleles were subsequently examined for altered *F. culmorum* susceptibility and all showed increased silique resistance, while leaf colonisation was reduced in two (*dmr1*-*3* and *dmr1*-*4*). Amino acid analysis of *dmr1* siliques revealed homoserine accumulation, which is undetectable in wild type plants. Exogenous application of L-homoserine reduced bud infection in both *dmr1* and wild type plants, whilst D-homoserine application did not. Delayed leaf senescence was also observed in *dmr1* plants compared to wild type and correlated with reduced *Fusarium* leaf colonisation.

**Conclusions:**

These findings suggest that common Arabidopsis *DMR1* mediated susceptibility mechanisms occur during infection by both obligate biotrophic oomycete and hemi-biotrophic fungal pathogens, not only in vegetative but also in reproductive plant tissues. This has the potential to aid the development of cereal crops with enhanced resistance to FEB.

**Electronic supplementary material:**

The online version of this article (doi:10.1186/s12870-014-0317-0) contains supplementary material, which is available to authorized users.

## Background

Fusarium Ear Blight (FEB) disease, also known as Fusarium head scab disease, is a globally significant threat to the floral tissues of small grain cereal crops such as wheat, barley and maize, caused by several species of ascomycete fungi of the genus *Fusarium*. The main causal agents of FEB in the UK are *Fusarium graminearum* and *F. culmorum* (Dean *et al*. [1], Goswami and Kistler [2], Parry *et al*. [3]). The disease can cause huge crop losses in epidemic years due to reduction in grain yield or grain quality and via the contamination of the grain with mycotoxins such as deoxynivalenol (DON) which make grain unsafe for human and animal consumption (Rocha *et al*. [4]). No commercially available wheat cultivars are fully resistant to FEB and the basis of resistance is complex and major quantitative trait loci (QTL) based (Bai and Shaner [5], Buerstmayr *et al*. [6], Jayatilake *et al*. [7], Zhou *et al*. [8]).

Both *F. graminearum* and *F. culmorum* infect the floral and silique tissue of *Arabidopsis thaliana* under experimental conditions, thereby providing a tractable model for the study of host pathogen interactions during FEB disease (Urban *et al*. [9]). A role for a number of Arabidopsis genes in resistance/susceptibility to Fusarium has been identified using this pathosystem (Cuzick *et al*. [10], Cuzick *et al*. [11], Makandar *et al*. [12], Makandar *et al*. [13], Savitch *et al*. [14], Van Hemelrijck *et al*. [15]). In addition, transgenic and chemical approaches have been used to alter Arabidopsis leaf and floral susceptibility to FEB causing *Fusarium* species (Asano *et al*. [16], Ferrari *et al*. [17], Kaur *et al*. [18], Koch *et al*. [19], Schreiber *et al*. [20]). These findings have the potential to improve defence against FEB in cereal crops using conventional and transgenic approaches, and also enhance our understanding of defence responses in plant reproductive tissues.

The Arabidopsis *downy mildew resistant* (*dmr*) mutants were isolated from a gain of function screen for resistance to the oomycete pathogen *Hyaloperonospora arabidopsidis*, following ethyl methanesulfonate (EMS) mutagenesis of plants of the susceptible genotype L*er*-0 harbouring the *enhanced disease susceptibility* mutation *eds1*-*2* (Van Damme *et al*. [21]). The *eds1*-*2* mutation in L*er*-0 has previously been shown not to alter the interaction outcome between *F. culmorum* and Arabidopsis floral or silique tissue (Cuzick *et al*. [11]). Of the five *dmr* mutant alleles identified, three (*dmr3*, *dmr4*, *dmr5*) showed constitutive expression of the salicylic acid mediated defence related gene *PR*-*1*. The remaining mutants, *dmr1* and *dmr6*, were mapped and identified as encoding mutations in the Arabidopsis homoserine kinase, and a putative 2-oxoglurarate oxygenase, respectively (van Damme *et al*. [22], van Damme *et al*. [23]). *DMR6* is associated with salicylic acid mediated defence signalling but is required for *H. arabidopsidis* susceptibility. Mutation of *dmr1* results in accumulation of homoserine in non-inoculated plants, and exogenous application of L-homoserine co-incident with *H. arabidopsidis* inoculation confers resistance in wild type plants. However, the precise role of L-homoserine in resistance is not known.

We used the Fusarium – Arabidopsis floral pathosystem (Urban *et al*. [9]) to assess the effects of the *dmr* mutations on *Fusarium* susceptibility in Arabidopsis floral, silique and rosette leaf tissue. Mutants *dmr1*-*1*, *dmr1*-*2*, *dmr5* and *dmr6* (all of which also carry the *eds1*-*2* mutation) were initially investigated; *dmr3* and *dmr4* have pleiotropic dwarf phenotypes which affect floral morphology and were therefore unsuitable for inclusion in this study. Here we present the novel finding that reduced function of the Arabidopsis homoserine kinase DMR1 confers resistance to *F. graminearum* and *F. culmorum* in siliques and/or reduces colonisation of rosette leaf tissues, with varying levels of resistance conferred by different *dmr1* mutant alleles. The siliques of *dmr1* plants accumulate homoserine but are not depleted in amino acids such as threonine and methionine which are downstream products of homoserine kinase activity. We also find that mutation of *DMR1* results in delayed leaf senescence which may relate to the observed reduced leaf colonisation phenotype. Exogenous application of L-homoserine reduces floral and silique disease severity in both *eds1*-*2* and *dmr1* plants, but does not inhibit *in vitro Fusarium* growth.

## Results

### A selection of the Arabidopsis downy mildew resistant mutants have altered susceptibility to *Fusarium culmorum* silique infection and rosette leaf colonisation

**Figure 1:**
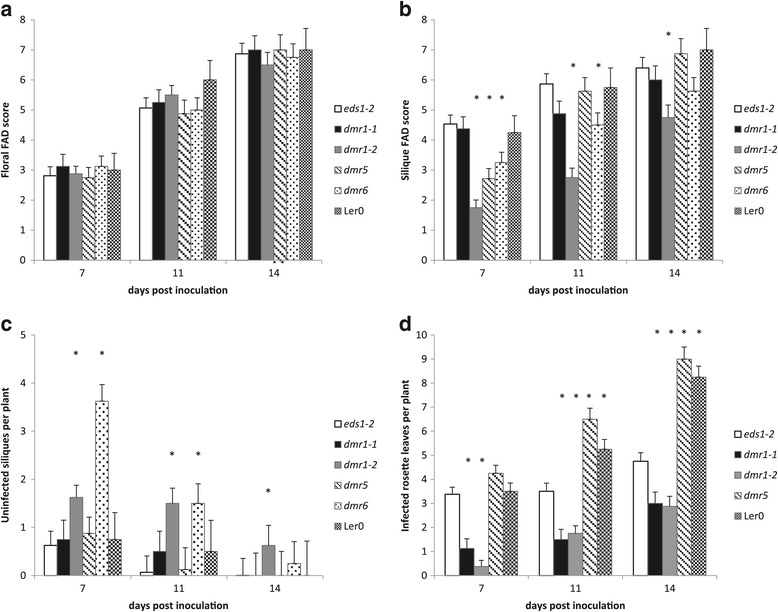
**Analysis of susceptibility to** ***F. culmorum*** **infection in four** ***downy mildew resistant*** **(** ***dmr*** **) mutant lines,** **compared to wild-** **type L** ***er-*** **0 and the parental genotype L** ***er-*** **0** ***eds1-*** ***2*** **.** Six plants per genotype were scored for **(a)** floral disease levels, **(b)** silique disease levels, **(c)** the number of healthy siliques, and **(d)** the number of infected rosette leaves, at 7, 11 and 14 days post inoculation (dpi). For the floral and silique evaluations the FAD- Fusarium-Arabidopsis Disease scoring system was used. Asterisks indicate genotypes significantly different from *eds1*-*2* at each time point (regression analysis followed by calculation of LSDs, p = <0.05). Error bars represent standard error of the mean. The experiment was repeated with similar results. Since the *dmr6* mutant flowers approximately 1 week later than *eds1*-*2*, *dmr6* plants were used in this experiment were 1 week older than those of other genotypes, and therefore rosette leaf data were not comparable due to increased senescence in the *dmr6* mutant.

**Figure 2:**
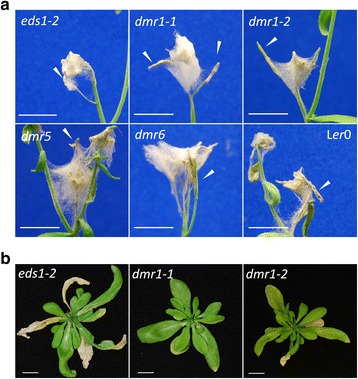
**Representative images of the floral and rosette leaf** ***Fusarium culmorum*** **infections of the Arabidopsis** ***downy mildew resistant*** **(** ***dmr*** **) mutants at 14 dpi,** **compared to the parental genotype** ***eds1*** ***-2*** **and wild type L** ***er-*** **0. (a)** Infected floral tissue of all genotypes. **(b)** Rosette leaves of the *dmr1* alleles compared to *eds1*-*2*. The stem and floral tissue has been removed from each plant in panel b. Bar =1 cm. White arrows = siliques with different levels of infection.

The Arabidopsis mutants *dmr1*-*1*, *dmr1*-*2*, *dmr5* and *dmr6*, which were generated in the L*er*-0 *eds1*-*2* background, were screened for altered susceptibility to *F. culmorum* infection compared to *eds1*-*2*. Wild type L*er*-0 was also included in the assay. Following spray inoculation with *F. culmorum* spores, the plants were scored for floral and silique disease levels, along with rosette leaf infection and number of uninfected green siliques, after 7, 11 and 14 days (Figures 1 and 2). There was no statistically significant difference in floral FAD (Fusarium-Arabidopsis Disease) score (Urban *et al*. [9]) between the genotypes tested (F_4, 93_ = 0.7, p = 0.591) at any of the time points assessed, with disease progressing at an equivalent rate in all genotypes (Figure 1a). At the time of inoculation, this tissue had been unopened green buds.

By contrast, there was a significant effect of genotype on silique FAD score (F_4, 91_ = 16.23, p = <0.01). The siliques assessed had been open flowers at the time of inoculation. The disease progression in the L*er*-0 and L*er*-0 *eds1*-*2* plants was identical (Figure 1b). The mutant allele *dmr1*-*2* had significantly reduced silique disease levels at all time points compared to *eds1*-*2* (Figure 1b, Figure 2a). This finding was confirmed in multiple independent experiments. Genotypes *dmr5* and *dmr6* had reduced silique disease symptoms at 7 and 11 days post inoculation (dpi) in the displayed experiment but these findings were not consistent across experiments. The *dmr1*-*2* plants had significantly higher numbers of uninfected green siliques than *eds1*-*2* at all time points, whilst for *dmr6* significantly more green siliques were observed at 7 and 11 dpi but not at 14 dpi (Figure 1c).

While healthy rosette leaf tissue is not susceptible to *Fusarium* infection under our experimental conditions, colonisation by the fungus occurs during leaf senescence. Colonisation of rosette leaves was formally assessed following the observation that *dmr1* leaves support less fungal growth. The number of colonised rosette leaves following the initial spray inoculation was found to be significantly affected by genotype (F_4, 93_ = 66.06, p = <.001). Both *dmr1* alleles had significantly fewer colonised rosette leaves than *eds1*-*2* at all time points (Figure 1d, Figure 2b). Interestingly, L*er*-0 had significantly more colonised rosette leaves per plant than *eds1*-*2*, indicating that the *eds1* mutation may have an effect on *F. culmorum* leaf susceptibility that was not identified in the previous study which focused on floral infection (Cuzick *et al*. [11]).

### Mutation of DMR1 reduces susceptibility to *F. graminearum*

**Figure 3:**
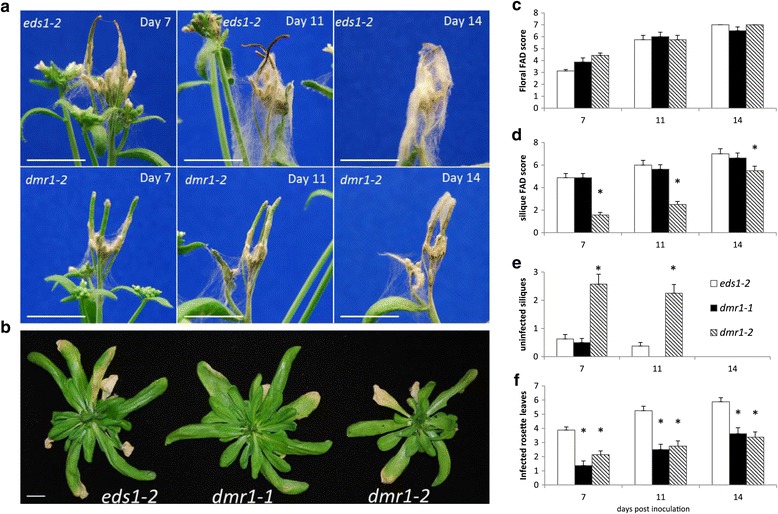
**Analysis of susceptibility to** ***F. graminearum*** **infection in plants harbouring different alleles of the** ***dmr1*** **mutation,** ***dmr1-*** ***1*** **and** ***dmr1-*** ***2,*** **compared to the parental line** ***eds1-*** ***2*** **. Panel (a)** shows infection of the apical inflorescence and siliques in *eds1*-*2* and *dmr1*-*2* at 7, 11, and 14 dpi. In **panel (b)** the rosette leaves of the *dmr1* alleles are compared to *eds1*-*2*, and the stem and floral tissues have been removed. Bar =1 cm. Eight plants per genotype were scored for **(c)** floral disease levels, **(d)** silique disease levels, **(e)** number of green, uninfected siliques and **(f)** number of infected rosette leaves and at 7, 11 and 14 dpi. Asterisks indicate genotypes significantly different from *eds1*-*2* at each time point (regression analysis followed by calculation of LSDs, p = <0.05). Error bars represent standard error of the mean. The experiment was repeated with similar results.

FEB disease is caused by several cereal infecting Fusaria species. Therefore, the susceptibility to *F. graminearum* infection was compared between the *dmr1* mutant alleles *dmr1*-*1* and *dmr1*-*2*, and the parental genotype *eds1*-*2* at 7, 11 and 14 dpi (Figure 3). Results were similar to those obtained for *F. culmorum*: No difference was observed in floral susceptibility (F_2, 62_ = 2.25, p = 0.114). Rosette leaf colonisation was affected by genotype (F_2, 62_ = 37.10, p < .001) with both *dmr1* alleles having fewer diseased rosette leaves than *eds1*-*2* (Figure 3b and f). Silique FAD scores and uninfected silique numbers also differed between genotypes (F_2, 62_ = 48.63 and 55.31 respectively, p = <.001). Silique FAD scores were lower in *dmr1*-*2* than *eds1*-*2* at all time points, with uninfected green siliques higher in *dmr1*-*2* at 7 and 11 dpi (Figure 3a, d and e). In these *F. graminearum* inoculated experiments, full infected and very necrotic siliques were visible in the *eds1*-*2* plants from 7 dpi onwards, whereas this extreme silique phenotype was rarely observed from 11 dpi onwards for either the *dmr1*-*1* or the *dmr1*-*2* plants. Overall these results indicate that both leaf and silique resistance conferred by mutation of *DMR1* is conserved across at least two cereal infecting *Fusarium* species.

### Multiple *dmr1* alleles have increased resistance to *F. culmorum*

**Figure 4:**
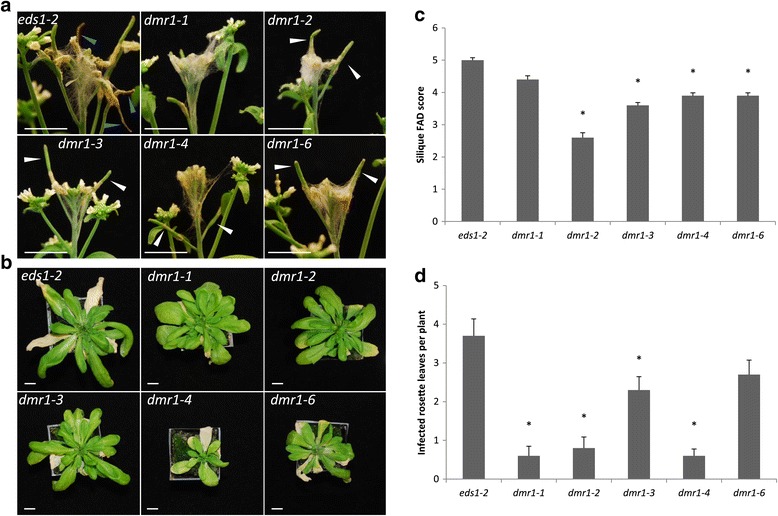
**Multiple** ***dmr1*** **alleles have reduced disease symptoms of** ***Fusarium culmorum*** **infection in siliques and rosette leaves.** Plants were spray inoculated with *F. culmorum* conidia at early flowering, and disease levels were assessed at 7 dpi. Representative images of apical inflorescences are shown at 7 dpi for **(a)** *eds1*-*2*, *dmr1*-*1*, *dmr1*-*2*, *dmr1*-*3*, *dmr1*-*4* and *dmr1*-*6*. Representative images of rosette leaves are shown at 13 dpi for **(b)** *eds1*-*2*, *dmr1*-*1*, *dmr1*-*2*, *dmr1*-*3*, *dmr1*-*4*, and *dmr1*-*6* –minus floral and stem tissues. Bar =1 cm. Blue arrow head – severely necrotic siliques visible in *eds1*-*2*. White arrow head – green siliques of *dmr1* plants. Silique FAD scores **(c)** and infected rosette leaves per plant **(d)** are shown at 7 dpi. Asterisks indicate genotypes significantly different from *eds1*-*2* (regression analysis followed by prediction of LSDs, p = <0.05). Error bars represent standard error of the mean. Data shown are pooled from two independent experimental replicates. *n* =10 (*eds1*-*2*, *dmr1*-*3*, *dmr1*-*4*, *dmr1*-*6*), *n* =5 (*dmr1*-*1*, *dmr1*-*2*).

In order to verify that the silique resistance phenotype observed in *dmr1*-*2* is a result of mutation of *DMR1* and not caused by a second EMS induced mutation, three additional alleles of *dmr1* (*dmr1*-*3*, *dmr1*-*4* and *dmr1*-*6*) were tested for altered resistance to *F. culmorum* (Figure 4). The *dmr1*-*2*, *dmr1*-*3*, *dmr1*-*4* and *dmr1*-*5* mutants all had lower silique disease levels than *eds1*-*2* (Figure 4a, c) (F_5, 49_ = 2.31, p = 0.005), whilst no differences in floral susceptibility were observed between the various *dmr1* genotypes and *eds1*-*2*. This again indicates that the open flowers and very immature siliques at the time of inoculation of the *dmr1* mutant plants were more resistant to *F. culmorum* infection than the green unopened buds. Fungal growth on rosette leaves was also different between genotypes (F_5, 49_ = 15.04, p = <0.001) with fewer rosette leaves per plant infected in genotypes *dmr1*-*1*, *dmr1*-*2*, *dmr1*-*3* and *dmr1*-*4* compared to *eds1*-*2* (Figure 4b, d). Collectively, these results confirm that increased silique and leaf resistance occurs in multiple *dmr1* alleles and is therefore likely a result of disruption of *DMR1* function.

Plants harbouring the mutant alleles *dmr1*-*1* and *dmr1*-*2* were also assessed for altered susceptibility to *F. culmorum* using a second inoculation method, namely the spore droplet, single silique point inoculation assay. This assay involves initially removing 1 mm of tissue from the tip of each immature silique and then placing the 1μl spore droplet onto the cut surface. No clear differences were seen in the distance of visible disease progression through the silique and pedicel between genotypes (Additional file 1: Figure S1).

### Homoserine accumulates in the siliques of *dmr1* mutant plants

**Figure 5:**
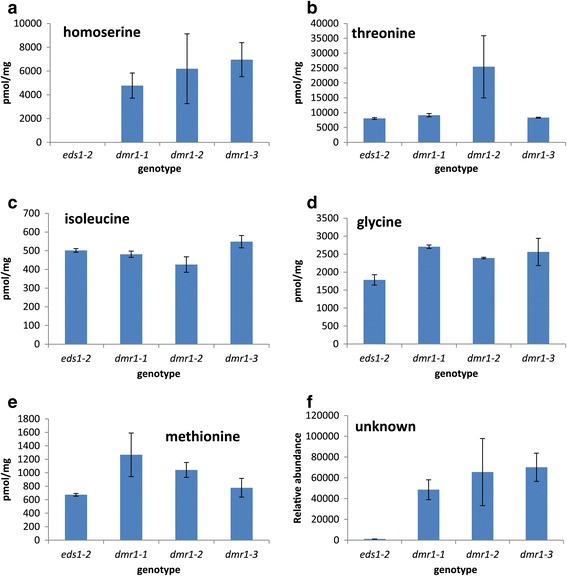
**Silique amino acid composition of three** ***dmr1*** **mutant alleles.** Gas chromatography mass spectroscopy (GC-MS) was used to identify and quantify the amino acids present in *dmr1*-*1*, *dmr1*-*2* and *dmr1*-*3* compared to *eds1*-*2* in the absence of Fusarium infection. Homoserine **(a)** was not detectable in *eds1*-*2* siliques but was abundant in the siliques of the *dmr1* mutants. Despite the absence of a functional homoserine kinase in the *dmr1* mutants, levels of threonine **(b)** isoleucine **(c)** glycine **(d)** and methionine **(e)**, downstream products of homoserine phosphorylation, were not reduced in the *dmr1* mutants compared to *eds1*-*2*. The level of an uncharacterised amino acid **(f)** was also elevated in all three *dmr1* mutants. Analysis was done on three independent biological silique samples per genotype. Bar = standard error.

Resistance of *dmr1* leaves to *H. arabidopsidis* infection was previously linked to elevated homoserine levels in 10 day old seedlings (van Damme *et al*. [23]). We analysed the amino acid composition of the siliques of three *dmr1* mutant alleles compared to *eds1*-*2* in order to identify whether homoserine also accumulates in *dmr1* siliques (Figure 5). Homoserine was not detectable in *eds1*-*2* siliques, but was abundant in *dmr1* siliques (Figure 5a). Homoserine levels were comparable between all three mutant alleles, but were higher on average in *dmr1*-*2* and *dmr1*-*3* siliques, which are resistant to *F. culmorum*, compared to *dmr1*-*1* siliques, which have wild type resistance levels. As previously observed in seedling tissue by van Damme and colleagues, mutation of homoserine kinase does not reduce levels of downstream amino acids (Figure 5b-e). Threonine levels were elevated in *dmr1*-*2* siliques, while methionine was more abundant in *dmr1*-*1* siliques. Interestingly, these changed levels observed in siliques correlate well with the levels of these amino acids in young seedlings. Glycine (which can be synthesised from threonine) was more abundant in all *dmr1* siliques than in *eds1*-*2*. An unidentified amino acid that is not a homoserine conjugate was also detected in *dmr1* samples but absent from *eds1*-*2*.

### Exogenous homoserine application reduces *F. culmorum* infection in Arabidopsis buds and siliques

Exogenous application of L-homoserine, but not D-homoserine, was previously shown to increase resistance in Arabidopsis and tomato to the obligate biotrophs *Hyaloperonospora arabidopsidis*, and *Oidium neolycopersici*, respectively, but homoserine did not inhibit spore germination or *in vitro* growth of oomycete pathogens (van Damme *et al*. [23], Huibers *et al*. [24]). We therefore investigated whether application of either enantiomer of homoserine affected growth of *Fusarium in vitro*, or had the ability to mimic the *dmr1* resistance phenotype *in planta*. We also investigated the effect of *in planta* threonine treatment on *Fusarium* growth, since threonine is elevated in some *dmr1* alleles and was previously shown to reduce *H. arabidopsidis* growth (Stuttmann *et al*. [25]).

No strong inhibitory effect of either homoserine isoform on *in vitro* growth was found for *F. culmorum* or *F. graminearum*, following 48 h incubation in synthetic nutrient poor media supplemented with L- or D-homoserine at concentrations ranging from 0 to 80 mM (Additional file 2: Figure S2).

**Figure 6:**
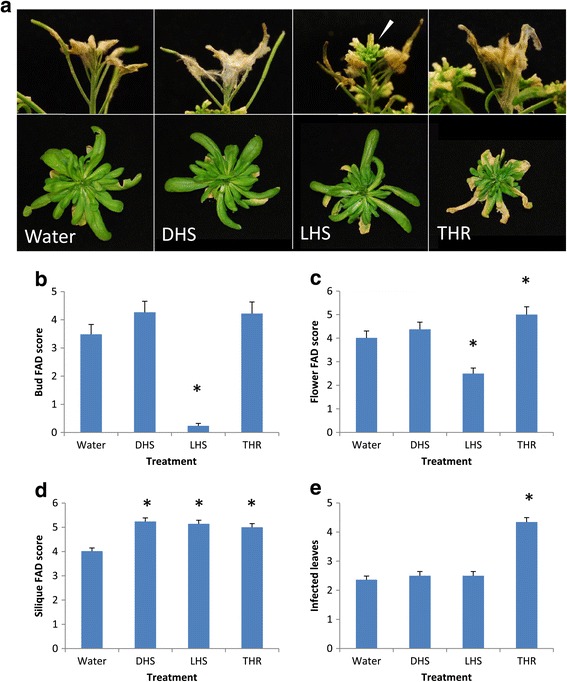
**The effect of exogenous amino acid treatments on** ***Fusarium*** **susceptibility in Arabidopsis floral,** **silique and rosette leaf tissue.** Arabidopsis plants of genotype *eds1*-*2* were sprayed at early flowering with either 10 mM D-homoserine (DHS), L-homoserine (LHS), threonine (THR) or sterile water, co-incident with *F. culmorum*. Amino acid/water treatments were then repeated daily for 6 dpi. Disease was assessed at 7 dpi. **a)** Images show infected apical inflorescences (upper panel) and rosette leaves (lower panel) – stem and floral tissue have been removed from rosettes. White arrow – green and opening buds present in LHS treated plants. Plants were scored for **(b)** bud disease, **(c)** open flower disease, **(d)** silique disease and **(e)** infected rosette leaf number. Asterisks indicate statistically significant differences from H_2_O treated plants (regression analysis followed by prediction of LSDs, p = <0.05, *n* = 8). Results are representative of two independent experiments.

To assess the *in planta* effects of amino acid treatment on *F. culmorum* growth, plants were sprayed with either 10 mM L- or D- homoserine (LHS, DHS), L-threonine (THR) or water, concurrent with spray inoculation with *F. culmorum* at early flowering. Amino acid/water treatments were repeated daily for 5 dpi. Significant differences in *F. culmorum* infection between treatments were found for unopened buds (F_3, 31_ = 41.38, p = <0.001), open flowers (F_3, 31_ = 7.31, p = <0.001), siliques (F_3, 31_ = 1.68, p = <0.001) and rosette leaves (F_3, 31_ = 7.71, p = <0.001). At 7 dpi, LHS treated buds showed little or no infection, and infection of opened flowers was also reduced, compared to DHS and water treated control plants (Figure 6a, b, c). Silique infection levels were slightly elevated in all amino acid treated plants compared to water controls in these experiments (Figure 6d). Threonine treatment increased *F. culmorum* colonisation in both open flowers and rosette leaves (Figure 6a and e).

Plants treated with threonine also exhibited leaf chlorosis and lesion formation in the absence of *F. culmorum* infection, indicating that threonine spray treatment at and above 10 mM may induce a cell death response. This result was consistent in both *eds1*-*2* and wild type L*er*-0 plants (Additional file 3: Figure S3).

**Figure 7:**
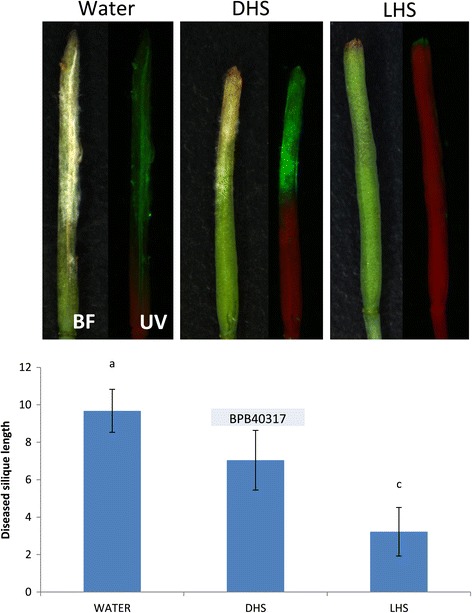
**Homoserine treatment reduces** ***F. culmorum*** **growth in point inoculated** ***eds1-*** ***2*** **siliques.** Tip-wounded *eds1*-*2* siliques were treated with 10 mM L-homoserine (LHS), D-homoserine (DHS) or sterile water coincident with *F. culmorum* inoculation. Amino acid/water treatment was repeated for 5 dpi. Images show infected siliques 8 dpi under bright field (BF) and UV light with a violet filter. Red fluorescence indicates healthy tissue, green fluorescence indicates infected tissue. The length of infection (mm) along three siliques per plant was assessed at 8 dpi. Different letters indicate statistically significant differences between treatments (analysis of variance followed by prediction of LSDs, p = <0.01, *n* = 12). Data were pooled from three independent experiments.

We also analysed the effect of D- and L-homoserine on *F. culmorum* infection of *eds1*-*2* siliques following single silique wound point inoculations (Figure 7). Siliques were droplet inoculated with water, DHS or LHS for 5 days following *F. culmorum* inoculation. There was a significant difference in *F. culmorum* infection development between treatments (ANOVA, p = <0.001). DHS treatment resulted in a modest reduction in *F. culmorum* growth along inoculated siliques compared to water treatment, while LHS treatment resulted in significantly less *Fusarium* growth than either water or DHS treatment, with most plants showing no externally visible infection. However, *F. culmorum* hyphae were present on and between seeds within some LHS treated siliques with externally uninfected pericarps (Additional file 4: Figure S4).

**Figure 8:**
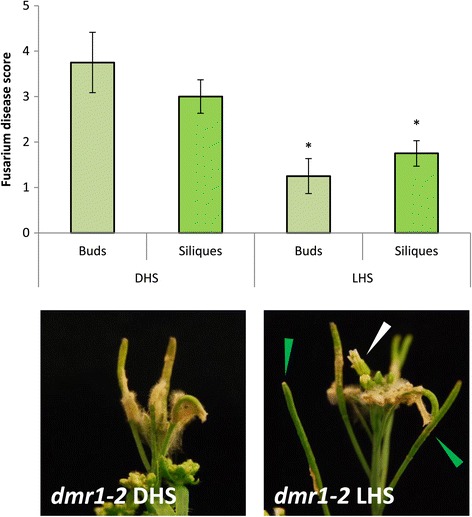
**Treatment of** ***dmr1-*** ***2*** **with LHS further reduces** ***Fusarium*** **silique susceptibility compared to DHS treated controls.** Arabidopsis plants of genotype *dmr1*-*2* were treated at early flowering with 10 mM L-homoserine (LHS) or D-homoserine (DHS) as a control, concurrent with *F. culmorum* spray inoculation. Amino acid/water treatments were repeated for 7 days. Bud and silique disease levels were assessed at 7 dpi. Asterisks indicate statistically significant differences between treatments for each tissue type (regression analysis followed by prediction of LSDs, p = <0.05, *n* = 8). Images show inoculated inflorescences 7 dpi. LHS treatment reduced bud and silique disease levels in *dmr1*-*2*. White arrow – opening uninfected buds. Green arrow – green uninfected siliques. Analysis based on pooled data from two independent experiments.

We also analysed the effect of LHS treatment on *dmr1* mutants using the spray treatment method (Figure 8). We found that exogenous LHS application, compared to DHS application, conferred *F. culmorum* resistance in *dmr1*-*2* buds (which are not resistant) equivalent to that seen in LHS treated *eds1*-*2* buds (Figure 6b). Furthermore, LHS treatment afforded a further increase in silique resistance in *dmr1*-*2* siliques, despite a high level of resistance already being conferred by the mutation.

### Mutation of DMR1 affects plant growth and senescence

During the growth of the experimental plants, *dmr1*-*2* plants appeared to be slightly smaller in size than *eds1*-*2* plants. Therefore, the rosette diameter and leaf number were measured and compared between 5-week old plants of genotypes *dmr1*-*1*, *dmr1*-*2* and *eds1*-*2*. The quantification of growth confirmed that the *dmr1*-*2* plants have approximately ~25% smaller rosettes on average than *eds1*-*2* (Additional file 5: Figure S5a & b), but that leaf number is similar between genotypes (Additional file 5: Figure S5c). This supports the recent findings by Huibers *et al*. [24] that some Arabidopsis *dmr1* mutants have reduced fresh weight compared to *eds1*-*2*. Leaf senescence between genotypes was also assessed by counting the number of visibly senescent leaves per plant at 7, 11, and 14 days after flowering (corresponding to assessment of infected plants at 7, 11 and 14 dpi), and found to be delayed in both *dmr1*-*1* and *dmr1*-*2* compared to *eds1*-*2* (Additional file 5: Figure S5d & e).

Silique number were compared between genotypes at 7, 11 and 14 days post flowering (corresponding to assessment of infected plants at 7, 11 and 14 dpi) to ensure that the increased number of uninfected siliques observed in *dmr1*-*2* was not due to more siliques being produced in this genotype. No difference was found between genotypes at any of the time points assessed (Additional file 5: Figure S5f). There was no evidence of increased silique number in the other *dmr1* alleles investigated in this study, although this was not formally assessed.

### Exogenous application of L-homoserine only partially and inconsistently affected Fusarium colonisation of wheat ears

The effect of exogenous homoserine application on Fusarium infection in wheat spikes was assessed by treating *F. graminearum* infected wheat spikelets with L-homoserine, D-homoserine or sterile water daily for 7 dpi. Fewer mean bleached spikelets and bent awns and higher grain number and weight were observed in L-homoserine treated plants compared to the other treatments in both experimental replicas. However, only the reduced number of bleached spikelets was statistically significant, and only in the first experimental replicate (Additional file 6: Figure S6) (p = 0.03).

## Discussion

In order to identify additional host genes controlling the outcome of the Fusarium–Arabidopsis interaction, as well as highlight components of defence signalling which are conserved in response to different pathogen types, we screened a number of *downy mildew resistant* mutants for altered susceptibility to the fungal pathogens *F. culmorum* and *F. graminearum*, which infect floral tissue in cereals and Arabidopsis. We identified that multiple loss of function mutant alleles of the Arabidopsis *HOMOSERINE KINASE* gene *DMR1* have increased resistance to *Fusarium culmorum*: Siliques of *dmr1*-*2*, *dmr1*-*3*, *dmr1*-*4* and *dmr1*-*6* were found to be more resistant to infection and leaves of *dmr1*-*1*, *dmr1*-*2*, *dmr1*-*3* and *dmr1*-*4*, supported reduced leaf colonisation. These phenotypes were also observed following *F. graminearum* infection of *dmr1*-*1* and *dmr1*-*2*.

These results indicate that there is potentially a common mechanism of susceptibility occurring in response to infection by both the downy mildew oomycete pathogen *H. arabidopsidis*, which is a leaf adapted obligate biotroph, and fungal hemi-biotrophic *Fusarium* species which are floral adapted. Mutation of At*DMR1* and its tomato ortholog *SlDMR1* has also recently been shown to increase resistance to the obligate biotrophic fungal mildew *Oidium neolycopersici* in an allele dependent manner, but has not been found to alter resistance to any other pathogens assessed, including the facultative biotrophic bacterium *Pseudomonas syringae* (van Damme *et al*. [23], Huibers *et al*. [24]). As with susceptibility to *Oidium neolycopersici* (Huibers *et al*. [24]), resistance to *Fusarium* in *dmr1* is allele dependent; while the *dmr1*-*1* allele supports less rosette leaf colonisation and has delayed senescence it does not confer resistance in siliques, despite homoserine accumulating within this tissue. One hypothesis is that homoserine accumulation caused by the *dmr1* mutation is not responsible for increased *Fusarium* silique resistance, and that DMR1 has an additional function contributing to silique susceptibility which is not abolished in the *dmr1*-*1* allele. Meanwhile the delayed leaf senescence and associated reduction in fungal colonisation in this and the other alleles is due to reduced homoserine kinase function, and is therefore conserved between alleles. However, this does not account for the reduction in disease afforded by exogenous application of homoserine. An alternative hypothesis is that the *dmr1*-*1* mutant harbours a second mutation as a result of the original EMS mutagenesis which mitigates the silique resistance phenotype, either by blocking the activity of the elevated homoserine or independently elevating silique susceptibility via another pathway. The latter would potentially be less likely to affect saprophytic colonisation of senesced leaf tissue. Further investigation is required to test these hypotheses.

Mutation of *DMR1* was not found to alter susceptibility to infection of unopened buds and young flowers, despite affecting both leaf and silique infection. Analysis of Arabidopsis *DMR1* expression using Genevestigator (Hruz *et al*. [26]) shows that *DMR1* is expressed at lower levels in some floral tissues than it is in vegetative tissue, namely in the stamens, anthers, stigma and sepals (Additional file 7: Figure S7a). Since homoserine kinase activity has been shown to be driven by homoserine accumulation (Lee *et al*. [27]), it may be that these tissues do not produce high levels of homoserine and are therefore unaffected by decreased DMR1 activity. Susceptibility of sepal and male reproductive tissue during early floral development pre-fertilisation may result in loss of the flower, whereas infection of these tissues post-pollination would have little effect on the siliques of resistant *dmr1* plants, as this tissue is shed during silique development.

Since *dmr1* induced resistance to *H. arabidopsidis* is proposed to be mediated by homoserine accumulation and can be mimicked by exogenous L-homoserine application in wild type plants, we investigated the effects of homoserine application on Fusarium growth *in vitro* and *in planta*. Treatment of *eds1*-*2* plants with L-homoserine (LHS) following spray inoculation with *F. culmorum* resulted in significantly decreased bud and flower colonisation by the fungus. This contrasts with the phenotype of *dmr1* mutants, which have increased silique and leaf, but not floral, resistance. As previously discussed, the Genevestigator analysis suggested that some floral organs may have lower HSK expression than other plant tissues (Additional file 7: Figure S7). This may result in longer persistence of the applied homoserine in buds than in siliques and other tissues, resulting in reduced fungal growth compared to other tissues. This was corroborated by the finding that exogenous L-homoserine application onto resistant *dmr1*-*2* siliques, which accumulate homoserine, further enhanced resistance to *F. culmorum* (Figure 8). We did find that more direct application of both the fungus and the LHS onto the tips of wounded siliques resulted in decreased fungal growth along the silique compared to water and DHS treated controls, indicating that homoserine treatment enhances resistance in wild type siliques under these infection conditions. However, in these experiments we also observed less *F. culmorum* infection following treatment with D-homoserine than with water, suggesting that the D-enantiomer of homoserine can also reduce Fusarium growth in certain situations. This finding contrasts with the results obtained following spray inoculation, where both amino acid forms increase silique disease. The biological reason for this is unclear. Formally, it is possible that daily application of distilled water to the silique tip might enhance the growth of Fusarium, which requires high humidity for infection, whereas the various osmotic properties of the amino acid solutions do not. Alternatively the D-homoserine may be eliciting a response from the wounded silique tip which then affects fungal growth. This demonstrates the importance of using the biologically inactive enantiomer as a control.

Exogenous application of homoserine onto the spikelets of *F. graminearum* infected wheat cultivar Apogee did not consistently reduce infection. The number of bleached spikelets was significantly lower in LHS treated plants (p = 0.03) at 10 days post inoculation in one experimental replica, but there was no significant difference in the degree of awn bending, grain number or grain weight. Therefore the effect of L-homoserine on Fusarium infection and disease development was less pronounced on wheat floral tissue compared to Arabidopsis. This interspecies difference is likely due to the rapid metabolism of the applied homoserine by the functional wheat homoserine kinase(s), preventing homoserine accumulation and activity. Future research should therefore focus on disruption of wheat homoserine kinase function.

This study also presents the novel finding that exogenous application of threonine induces host cell death in Arabidopsis leaves and increases *F. culmorum* colonisation. This raises further questions about the effects of amino acid metabolism on plant defence against different pathogen species and lifestyles. van Damme and colleagues did not find an effect of exogenous threonine application on *H. arabidopsidis* susceptibility when amino acids were applied by vacuum infiltration. However, Stuttmann *et al*. [25] found that spray application of 1-5 mM threonine resulted in decreased *H. arabidopsidis* sporulation in L*er*-0 *eds1*-*2* plants. These contrasting outcomes are interesting. *H. arabidopsidis* is a classic obligate biotroph and would therefore be sensitive to any host induced cell death which would limit this pathogen’s access to living tissue. By contrast *Fusarium* has been shown to have a switching *in planta* lifestyle with host cell death an integral feature of the later disease formation process (Brown *et al*. [28], Desmond *et al*. [29], Thaler *et al*. [30]). Cereal infecting Fusaria are also able to colonise saprophytically dead plant tissue. Threonine mediated chlorosis may therefore facilitate *Fusarium* colonisation while preventing growth of obligate biotrophic pathogens.

Related to this is the finding that mutation of *DMR1* results in delayed senescence. Analysis of *DMR1* expression during plant development using GENEVESTIGATOR (Hruz et al. [26]) shows that expression is fairly static throughout plant development but increases during senescence (Additional file 7: Figure S7b). This suggests that DMR1 function could have a role in programmed cell death and senescence. The delayed DMR1 dependent cell death in the *dmr1* mutants may restrict Fusarium disease progression and prevent its successful exploitation of host cell death (Thaler *et al*. [30]). For pathogens such as *Fusarium* with a combined hemi-biotrophic and saprophytic life style strategy, delayed cell death could prevent full tissue exploitation and the gaining of additional nutrition from the cellular debris. This is supported by the finding that *dmr1* leaves display delayed senescence accompanied by a decrease in *Fusarium* colonisation. However, the delayed cell death may not be the underlying cause of the enhanced resistance. For example, the manner in which delayed cell death might help protect plants against obligate biotrophic pathogens such as *H. arabidopsidis* is not clear. It is formally possible that the normal amino acid ratios found in healthy Arabidopsis tissue are modified in the *dmr1* mutants and this alters the efficiency of nutrient acquisition via the haustoria interfaces in obligate biotrophic interactions as well as altering the switching lifestyle of hemibiotrophic pathogens. In this regard, the identification of the novel accumulating amino acid in the three *dmr* mutants, but not *eds1*-*2* (Figure 5) remains a priority.

Huibers *et al*. [24] found a correlation between reduced Arabidopsis plant fresh weight in different *dmr1* mutant alleles and the level of resistance conferred to *O. neolycopersici*. The authors concluded that it might be difficult to obtain *dmr1* alleles in crop species which conferred enhanced resistance to this pathogen in the absence of a fitness cost. While the *dmr1*-*3* mutant allele did not confer a significant growth penalty, likewise it did not confer resistance to *O. neolycopersici*. However, in the current study, *dmr1*-*3* conferred resistance to *F. culmorum* in both the leaf and silique tissue of Arabidopsis, although the leaf phenotype was not as strong as in other alleles. Investigation into the effects of homoserine kinase disruption in FEB-susceptible cereal crops is therefore warranted. This may be achieved both through stable transgenesis and the use of an inducible promoter to drive an RNAi construct. Alternatively, the use of transient Virus Induced Gene Silencing (Lee *et al*. [31], Lee *et al*. [32]) of cereal homoserine kinases could be deployed.

In this study and that of van Damme *et al.* [21] it was found that *dmr1* plants have wild type or elevated levels of the amino acids methionine, threonine and isoleucine, in both foliar and silique tissue (Figure 5). These amino acids are understood to be synthesised directly via the activity of homoserine kinase. The abundance of these three amino acids in plants with severely reduced homoserine kinase function therefore challenges the current understanding of amino acid biosynthetic pathways. It may be the case that these amino acids are being synthesised via alternative, currently unidentified pathway(s) in the *dmr1* mutants, which are independent of homoserine kinase. Alternatively, mutated homoserine kinase may retain some residual function: Homoserine is synthesised via the activity of Arabidopsis aspartate kinases, which are negatively regulated by accumulation of S-adenosylmethionine (SAM), synthesised from methionine (Curien *et al.* [33,34]). Reduced methionine biosynthesis may therefore result in increased aspartate kinase activity, shunting more homoserine into the pathway. Some of the accumulating homoserine might then be phosphorylated by the mutated homoserine kinase, restoring equilibrium in the pathway. However, no changes were observed in aspartate levels between wild type and *dmr1* mutant plants. Experiments to compare aspartate kinase expression and activity between genotypes would be informative.

## Conclusions

This study has identified that a series of mutations in the Arabidopsis homoserine kinase gene *DMR1* confers resistance in silique tissue to the primary causal agents of cereal FEB disease, a source of crop yield losses and grain contamination. Siliques of the *dmr1* mutants accumulate homoserine, and exogenous application of L-homoserine confers resistance to the floral and silique tissues of both mutant *dmr1* and wild type *DMR1* plants. These finding offer the possibility of developing a novel source of resistance to an economically important floral crop disease for which few other resistance mechanisms exist. Further work will use virus induced gene silencing of the wheat *DMR1* ortholog to explore the potential of homoserine in *Fusarium* resistance in wheat. However, the mechanism by which homoserine accumulation in plant tissue mediates resistance is still not fully understood, and may be key to fully exploiting *dmr1* based resistance which has the potential for use in multiple crop species.

## Methods

### Plant growth

The Arabidopsis *downy mildew resistant* mutant collection and parent genotype *eds1*-*2* were a gift from Guido van den Ackerveken, Utrecht University, The Netherlands. Ecotype L*er*-0 (NASC stock NW20) was a gift from Graham McGrann, John Innes Centre, UK. Plants were grown in Levingtons F2 + S compost in a Fitotron® ‘walk in’ plant growth chamber (www.fitotron.co.uk), with a 16 h light/ 8 h dark cycle at temperatures of 20°C (light) and 17°C (dark), with 150 μmol m^−2^ s^−1^ fluorescent illumination, at 70% humidity.

### Fusarium growth and storage

*F. culmorum* strain 98/11 (NRRL 54112) and *F. graminearum* strain PH-1 (NRRL 31084) were propagated as previously described (Urban *et al*. [9]; Cuzick *et al*. [10]); Conidia were transferred from frozen stocks stored at -80°C onto synthetic nutrient poor agar plates (SNA -0.1% KH_2_PO_4_, 0.1% KNO_3_, 0.1% MgSO_4_x7 H_2_O, 0.05% KCl, 0.02% glucose, 0.02% saccharose, 2% agar) for 8-11 days, and then transferred onto potato dextrose agar (PDA) plates for 48 h to encourage high levels of conidial production. Conidia were then suspended in sterile distilled water and filtered through sterile Miracloth (Calbiochem®), and stored at -80°C prior to plant inoculations. Studies using *F. graminearum* strain PH-1 were conducted under PHSI license 101948/198285/2.

### Arabidopsis-Fusarium infection assays

For spray inoculation assays, plants were selected at early flowering (2-4 open flowers per plant) and the whole plant spray inoculated with *F. culmorum* or *F. graminearum* conidia suspended in sterile water at a concentration of 10^6^ conidia/ml. Control plants were spray inoculated with sterile water.

For amino acid treatment studies, inoculated plants were sprayed daily with a solution of 10 mM L-homoserine, D-homoserine or L-threonine, or sterile water as a control. This was repeated daily for 5 days post inoculation.

For single silique wound point inoculations, plants with six young siliques were selected for inoculation. The top 1 mm of each silique was removed using sterile scissors and the tip inoculated with a 1 μl droplet containing approx. 10^5^*Fusarium* conidia suspended in sterile H_2_O. Sterile H_2_O alone was used as a control. For amino acid treatments, conidial suspensions were supplemented at the time of inoculation with 20 mM L-homoserine or D-homoserine, or sterile water as a control. Amino acid suspensions were then re-applied for 6 days post inoculation.

Plants were transferred to inoculation boxes at 100% relative humidity for the duration of the experiment and kept in darkness for 20 h following inoculation.

### Scoring of Fusarium disease symptoms

Plants were scored for floral and silique disease symptoms using the Fusarium-Arabidopsis disease (FAD) scoring system devised by Urban *et al*. [9]. Disease scores per plant were assigned from 0 (no disease symptoms) to 7 (severe disease symptoms with constriction of the main stem) for apical flowers and siliques (Additional file 8: Table S1). The silique score applies to the most diseased silique on each plant, only increasing if all siliques display equivalent disease, and does not therefore take account of the number of siliques escaping disease. For this reason, uninfected green siliques per plant were also counted. The number of diseased rosette leaves per plant was also counted. At least five plants per treatment or genotype were used in each experiment, and each experiment was done at least twice.

### *In vitro* Fusarium growth tests

*F. culmorum* and *F. graminearum* conidia at a concentration of 2x10^5^ conida ml^-1^ were cultured for two days in 96 well flat bottomed culture plates in 200 μl synthetic nutrient poor liquid media supplemented with either L- or D-homoserine at a concentration range from 0 to 80 mM. Absorbance as a surrogate for fungal growth was measured for each homoserine concentration as previously described (Fan *et al*. [35]). Three biological replicates were included per fungal isolate/amino acid treatment, and the experiment repeated.

### Wheat infection and amino acid treatment

The dwarf wheat cultivar Apogee (Bugbee, Koerner *et al*. [36]) was used for wheat infection assays. The 8th and 9th spikelets of ears at anthesis were point inoculated with 5 μl of *F. graminearum* conidial suspension at 10^5^ ml^-1^. In addition, the 6th-11th spikelets were treated with 5 μl of either 10 mM L-homoserine or D-homoserine, or sterile water. The three different treatments were then repeated daily for 7 days. The number of bleached spikelets and bent awns (preceding bleaching in infected spikelets) was assessed, and grain weight and number recorded at 10 dpi following dissection of the rachis, as per Baldwin *et al*. [37].

### Analysis of silique amino acids

The amino acid content of 15 mg freeze dried and ground silique samples from *dmr1* mutant and *eds1*-*2* plants was analysed using the EZFaast GC-MS physiological amino acid analysis kit, according to the manufacturers’ instructions. The protocol was amended such that the addition of the internal standard supplied with the kit was omitted and the final solvent evaporation step with reconstitution in organic solvent was replaced with a 1:10 dilution with the organic solvent (reagent 6). Samples were analysed on an Agilent 5975 Inert MSD coupled to a 7890A Gas Chromatograph fitted with a Zebron Amino acid ZB-AAA column (10 m × 0.25 mm I.D. Phenomenex, Cheshire, UK), Gestel MPS2 autosampler and split/splitless injector (fitted with quartz wool packed SGE FocusLiner). For each genotype three biological replicates were analysed, each consisting of siliques from ~8 pooled 6-week old plants. The internal standard, amino acid standard solutions and glutamine standard were obtained from Sigma (Dorset, UK). Homoserine standard was obtained from Koch-Light Laboratories, Colnworth, Bucks, UK.

### Microscopy

Fusarium infected Arabidopsis siliques were imaged using a Leica M205 FA stereomicroscope and accompanying LAS-AF6000 software, using white light or UV light with a Violet filter (Excitation: 425/40 nm, Emission: 475 nm).

### Statistical analysis

The Arabidopsis – Fusarium disease susceptibility data were subjected to regression analysis fitted to a general linear model with assumed Poisson distribution. For mutant experiments with multiple time points, the effects of genotype and time, and the interactions between genotype and time, were examined. Where a significant effect of genotype or treatment was found (p = <0.05), genotype/treatment means and least significant differences (LSDs) between genotypes or treatments were calculated at a 5% confidence level and means for all genotypes compared to genotype *eds1*-*2*, and all amino acid treatments compared to water treatment, to identify significant differences. In the absence of an interaction between genotype and time, one mean per genotype was predicted by amalgamating data from all time points, with corresponding LSDs. Where a significant interaction between genotype and time was observed, means and LSDs to *eds1*-*2* were calculated for each genotype at each time point assessed. For analysis of the effect of LHS on *dmr1*-*2* compared to *eds1*-*2* LSDs between each genotype/treatment combination were used to identify statistically significant differences between treatments. For single silique point wound inoculations, analysis of variance (ANOVA) was used to compare fungal growth (in mm) along the silique. LSDs (p = 0.01) were calculated between treatments. All statistical analysis was done using Genstat v16 (Payne *et al*. [38]).

### Supporting data

All relevant supporting data can be found within the supplementary files accompanying to this article.

### Arabidopsis mutant loci

The following Arabidopsis loci are associated with this study: *dmr1*; AT2G17265, *dmr6*; AT5G24530, *eds1*-*2*; AT3G48090. Further information can be obtained from www.arabidopsis.org.

## Additional files

Additional file 1: Figure S1. Images of individual siliques point inoculated at the cut tip. (a-c) Water inoculated controls show comparable development and seed set between *eds1-2* and *dmr1-1* and *dmr1-2* genotypes. (d-i) Comparable levels of *Fusarium culmorum* infection of *dmr1* mutant and *eds1-2* siliques 7 days post inoculation. In panels d through f, whole infected split (left) and intact (right) siliques are shown. In panels g through i are close-up images of infected seeds. Shown are representative images present in multiple biological replicates. Additional file 2: Figure S2. Homoserine does not inhibit Fusarium hyphal growth *in vitro*. Spores of either *F. culmorum* or *F. graminearum* were cultured for 2 days in synthetic nutrient poor media supplemented with (a, b) D-homoserine and (c, d) L-homoserine at concentrations ranging from 0 to 80 mM. Graphs show the optical density at 600 nm of fungal colonies after 2 days growth. The experiment was repeated with similar findings. Additional file 3: Figure S3. Threonine (THR) mediated chlorosis in rosette leaves of Arabidopsis genotypes L*er*-0 and *eds1-2*. Plants were sprayed with 10 mM threonine or water daily for 5 days, first treatment coincident with *F. culmorum* or mock (water) spray inoculations. The effect of threonine was most pronounced in Fusarium inoculated leaves. Threonine from two different commercial suppliers was tested with identical outcomes. Additional file 4: Figure S4. Homoserine treatment reduces *F. culmorum* growth in point inoculated *eds1-2* siliques. Tip-wounded *eds1-2* siliques were treated with 10 mM L-homoserine (LHS), D-homoserine (DHS) or sterile water coincident with *F. culmorum* inoculation. Amino acid/water treatment was repeated for 5 dpi. Images show opened silique sections at 8 dpi. Tissue necrosis and fungal growth is evident in the pericarp (P) and seed (S) of water and D-homoserine (DHS) treated siliques. L-homoserine (LHS) treated siliques have predominantly uninfected pericarps, but some externally uninfected LHS treated siliques revealed, when opened, the presence of fungal colonisation within the silique (far right). Additional file 5: Figure S5. Differences in developmental morphology and senescence between the *dmr1-1* and *dmr1-2* mutant alleles and *eds1-2*. (a, b) Rosette diameter is reduced in 5-week old plants of genotype *dmr1-2* compared to *eds1-2*. (c) Leaf number is comparable between genotypes. (d, e) Leaf senescence is delayed in both *dmr1-1* and *dmr1-2*. Panel d shows the appearance of the rosettes of flowering plants at 14 days post flowering. (f) Silique number was equivalent between all genotypes throughout seed set. These phenotypes were observed across multiple experimental replicates. Asterisks indicate significant difference from *eds1-2*. *p < 0.05, **p = <0.01 (b – ANOVA, e – Regression analysis). Additional file 6: Figure S6. Effect of L-homoserine application on Fusarium infection of wheat. Spikes of wheat cultivar Apogee were point inoculated with *F. graminearum* and then treated with either L-homoserine (LHS) D-homoserine (DHS) or water for 7 days. The number of bent awns (a) and bleached spikelets (b) along with grain weight (c) and number (d) per plant were assessed at 10 dpi. (*) The number of bleached spikelets was significantly lower in LHS treated plants (p = 0.03). No statistically significant difference between treatments was found for other parameters (ANOVA, p = >0.05). Bar = standard error. Additional file 7: Figure S7. GENEVESTIGATOR analysis of the expression profile of Arabidopsis *DMR1*. A) Tissue specific expression levels across different floral tissues. B) Development stage specific expression levels. Additional file 8: Table S1. Scoring of Fusarium disease in Arabidopsis floral and silique tissue, adapted from Urban et al. [9]. Plants were given separate scores for floral and silique infection from 0 (no disease) to 7 (constriction of the main stem). The intermediate scores of 2 and 4 (F), and 2, 4 and 6 (S) were reserved for when all the tissue on a single plant exhibited the disease phenotype described for the preceding score.
