# Supplementary material for: Mutations in the Arabidopsis homoserine kinase gene DMR1 confer enhanced resistance to Fusarium culmorum and F. graminearum
Source: BMC Plant Biol. 2014 Nov 29;14:317. doi: 10.1186/s12870-014-0317-0 (PMC4258817; doi:10.1186/s12870-014-0317-0)
Supplement: Additional file 1: Figure S1. — Images of individual siliques point inoculated at the cut tip. (a-c) Water inoculated controls show comparable development and seed set between eds1-2 and dmr1-1 and dmr1-2 genotypes. (d-i) Comparable levels of Fusarium culmorum infection of dmr1 mutant and eds1-2 siliques 7 days post inoculation. In panels d through f, whole infected split (left) and intact (right) siliques are shown. In panels g through i are close-up images of infected seeds. Shown are representative images present in multiple biological replicates. [file 12870_2014_317_MOESM1_ESM.pptx]

## Slide 1
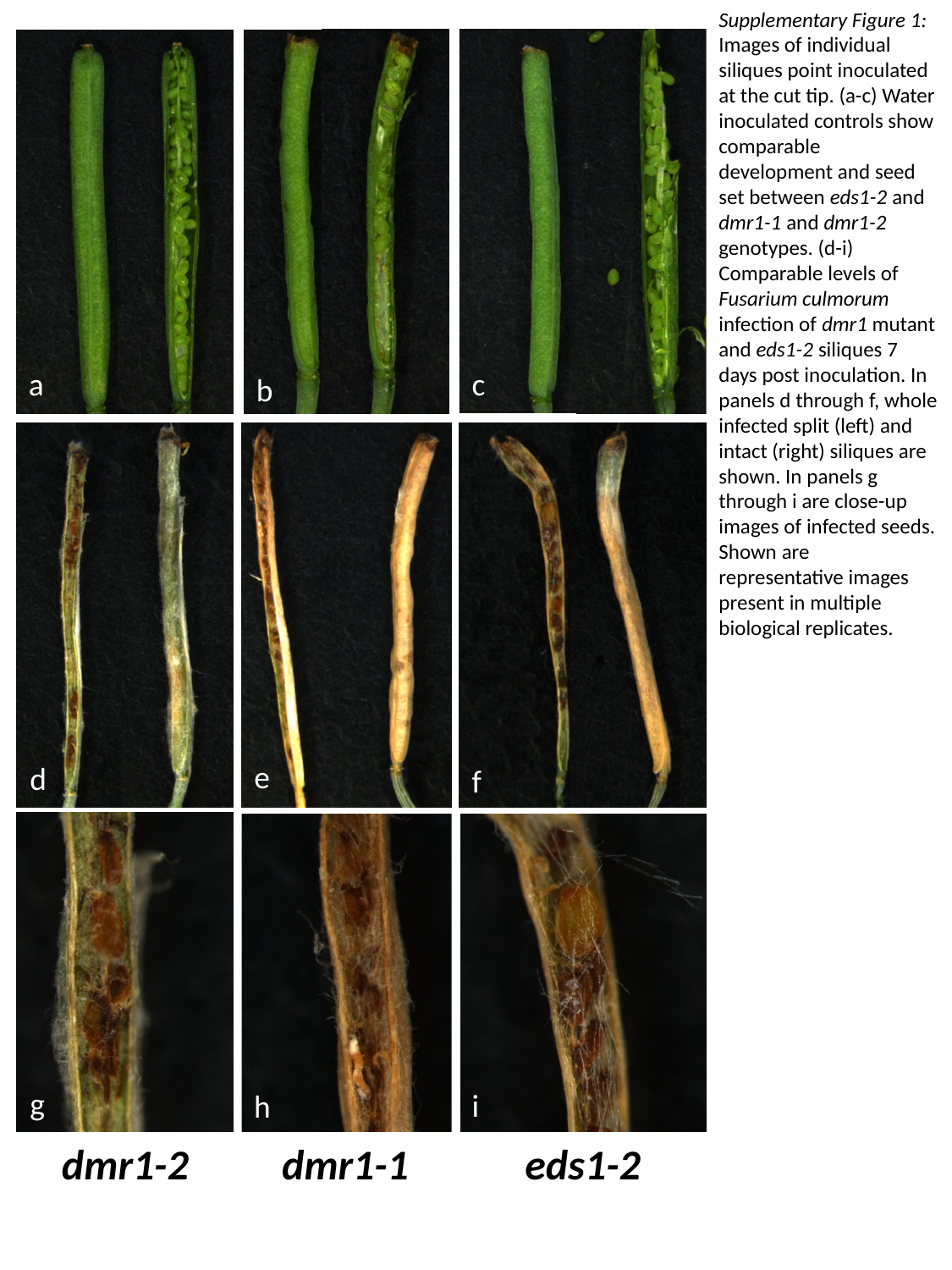

Supplementary Figure 1: Images of individual siliques point inoculated at the cut tip. (a-c) Water inoculated controls show comparable development and seed set between eds1-2 and dmr1-1 and dmr1-2 genotypes. (d-i) Comparable levels of Fusarium culmorum infection of dmr1 mutant and eds1-2 siliques 7 days post inoculation. In panels d through f, whole infected split (left) and intact (right) siliques are shown. In panels g through i are close-up images of infected seeds. Shown are representative images present in multiple biological replicates.
a
c
b
e
d
f
g
i
h
dmr1-2
dmr1-1
eds1-2
