# Supplementary material for: Mutations in the Arabidopsis homoserine kinase gene DMR1 confer enhanced resistance to Fusarium culmorum and F. graminearum
Source: BMC Plant Biol. 2014 Nov 29;14:317. doi: 10.1186/s12870-014-0317-0 (PMC4258817; doi:10.1186/s12870-014-0317-0)
Supplement: Additional file 2: Figure S2. — Homoserine does not inhibit Fusarium hyphal growth in vitro. Spores of either F. culmorum or F. graminearum were cultured for 2 days in synthetic nutrient poor media supplemented with (a, b) D-homoserine and (c, d) L-homoserine at concentrations ranging from 0 to 80 mM. Graphs show the optical density at 600 nm of fungal colonies after 2 days growth. The experiment was repeated with similar findings. [file 12870_2014_317_MOESM2_ESM.pptx]

## Slide 1
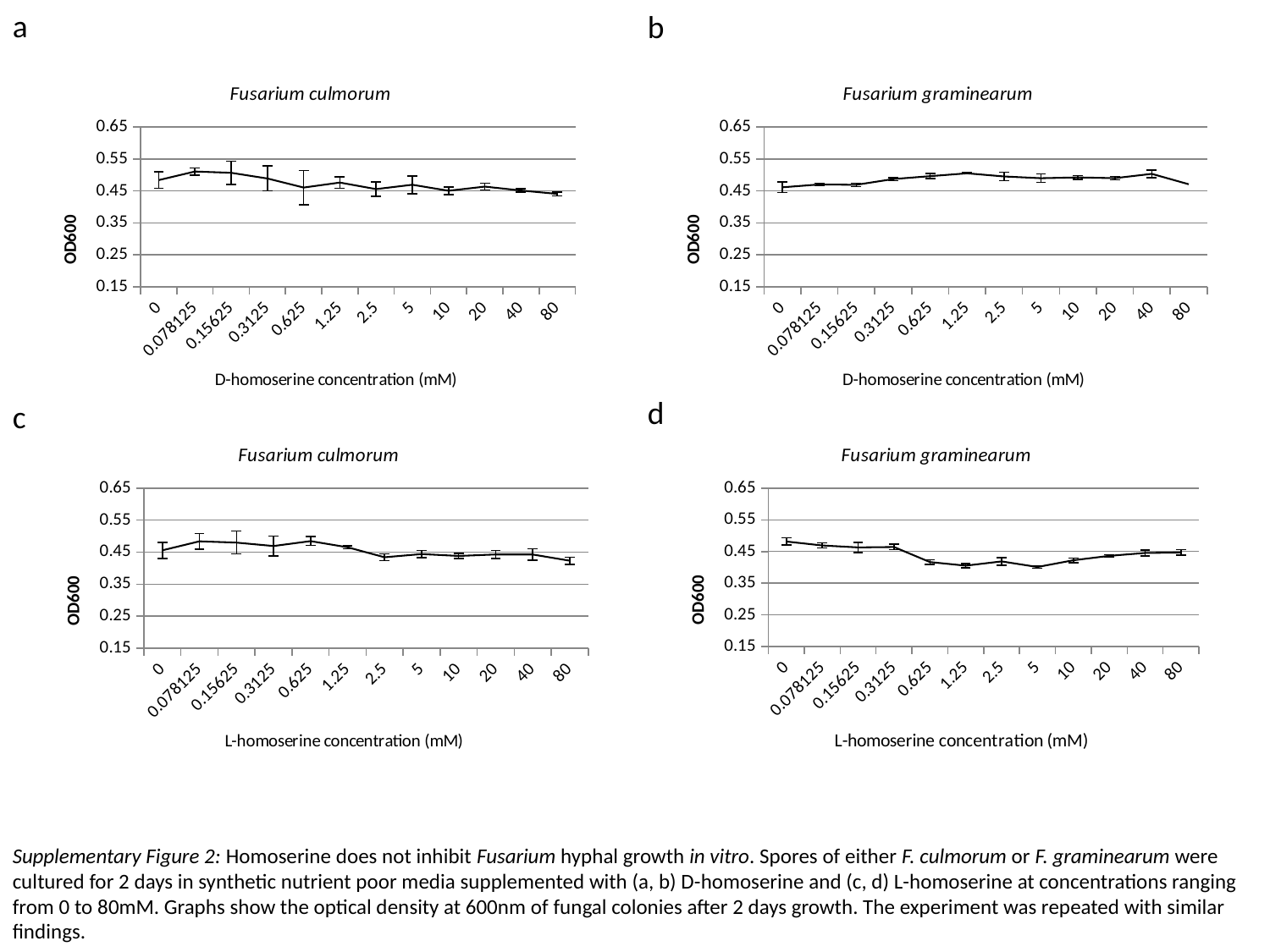

a
b
### Chart: Fusarium culmorum
| Category | |
|---|---|
| 0 | 0.4838625 |
| 7.8125E-2 | 0.510779166666667 |
| 0.15625 | 0.506614583333333 |
| 0.3125 | 0.488904166666667 |
| 0.625 | 0.460491666666667 |
| 1.25 | 0.476110416666667 |
| 2.5 | 0.4556375 |
| 5 | 0.4693125 |
| 10 | 0.4504875 |
| 20 | 0.463441666666667 |
| 40 | 0.451252083333333 |
| 80 | 0.4407125 |
### Chart: Fusarium graminearum
| Category | |
|---|---|
| 0 | 0.461375 |
| 7.8125E-2 | 0.470304166666667 |
| 0.15625 | 0.46915 |
| 0.3125 | 0.487089583333333 |
| 0.625 | 0.49616875 |
| 1.25 | 0.5051125 |
| 2.5 | 0.49520625 |
| 5 | 0.489727083333333 |
| 10 | 0.492208333333333 |
| 20 | 0.489939583333333 |
| 40 | 0.503325 |
| 80 | 0.470895833333333 |d
c
### Chart: Fusarium culmorum
| Category | |
|---|---|
| 0 | 0.455902083333333 |
| 7.8125E-2 | 0.48394375 |
| 0.15625 | 0.479914583333333 |
| 0.3125 | 0.469314583333333 |
| 0.625 | 0.484541666666667 |
| 1.25 | 0.465295833333333 |
| 2.5 | 0.434170833333333 |
| 5 | 0.444120833333333 |
| 10 | 0.438247916666667 |
| 20 | 0.442791666666667 |
| 40 | 0.442672916666667 |
| 80 | 0.423308333333333 |
### Chart: Fusarium graminearum
| Category | 0.482 0.469 0.463 0.464 0.417 0.405 0.419 0.401 0.422 0.436 0.445 0.447 |
|---|---|
| 0 | 0.48168125 |
| 7.8125E-2 | 0.469208333333333 |
| 0.15625 | 0.463025 |
| 0.3125 | 0.464229166666667 |
| 0.625 | 0.416729166666667 |
| 1.25 | 0.4054125 |
| 2.5 | 0.418808333333333 |
| 5 | 0.401208333333333 |
| 10 | 0.4224125 |
| 20 | 0.436320833333333 |
| 40 | 0.445439583333333 |
| 80 | 0.447002083333333 |Supplementary Figure 2: Homoserine does not inhibit Fusarium hyphal growth in vitro. Spores of either F. culmorum or F. graminearum were cultured for 2 days in synthetic nutrient poor media supplemented with (a, b) D-homoserine and (c, d) L-homoserine at concentrations ranging from 0 to 80mM. Graphs show the optical density at 600nm of fungal colonies after 2 days growth. The experiment was repeated with similar findings.
