# Supplementary material for: Mutations in the Arabidopsis homoserine kinase gene DMR1 confer enhanced resistance to Fusarium culmorum and F. graminearum
Source: BMC Plant Biol. 2014 Nov 29;14:317. doi: 10.1186/s12870-014-0317-0 (PMC4258817; doi:10.1186/s12870-014-0317-0)
Supplement: Additional file 3: Figure S3. — Threonine (THR) mediated chlorosis in rosette leaves of Arabidopsis genotypes Ler-0 and eds1-2. Plants were sprayed with 10 mM threonine or water daily for 5 days, first treatment coincident with F. culmorum or mock (water) spray inoculations. The effect of threonine was most pronounced in Fusarium inoculated leaves. Threonine from two different commercial suppliers was tested with identical outcomes. [file 12870_2014_317_MOESM3_ESM.pptx]

## Slide 1
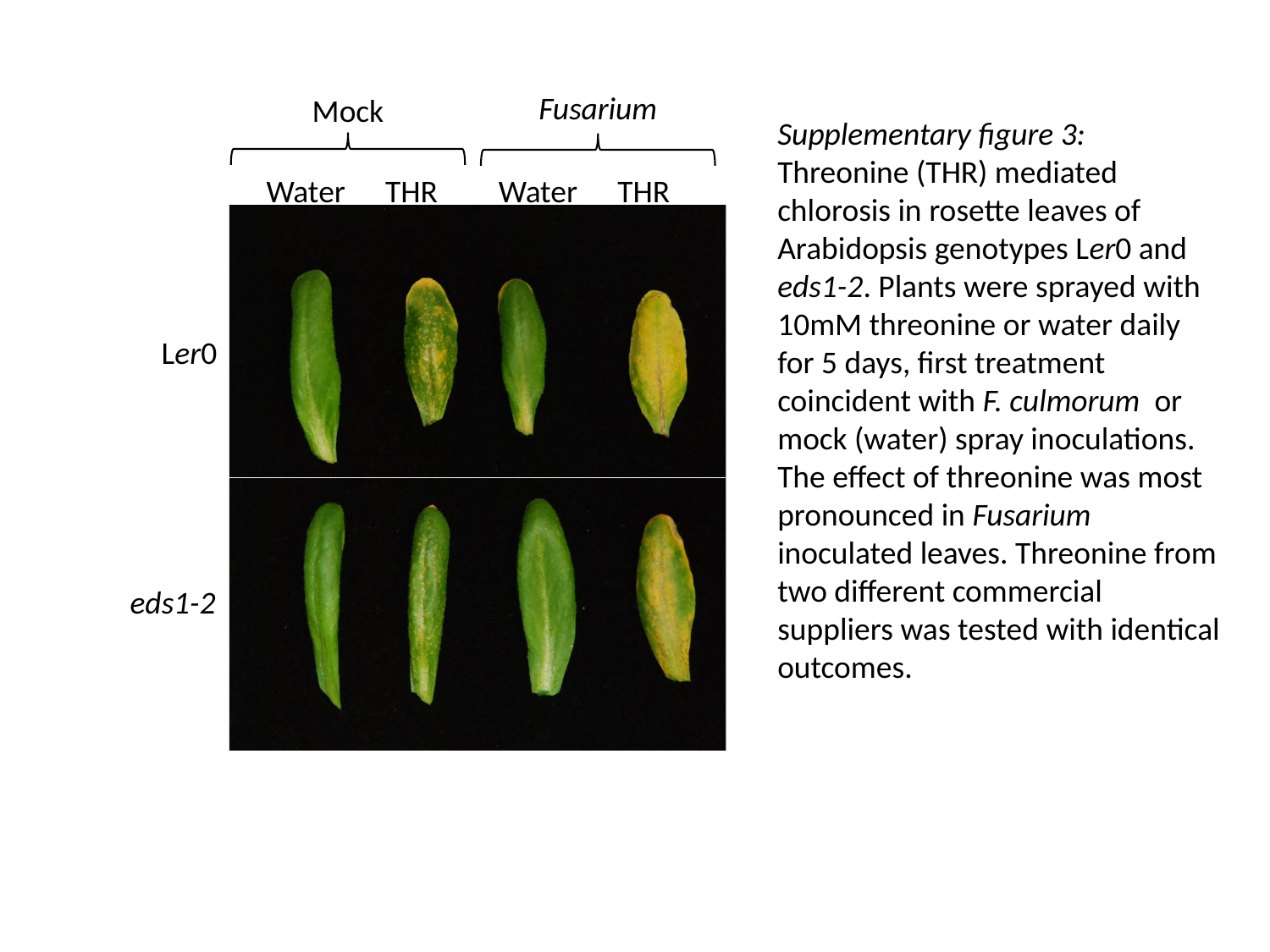

Fusarium
Mock
Supplementary figure 3: Threonine (THR) mediated chlorosis in rosette leaves of Arabidopsis genotypes Ler0 and eds1-2. Plants were sprayed with 10mM threonine or water daily for 5 days, first treatment coincident with F. culmorum or mock (water) spray inoculations. The effect of threonine was most pronounced in Fusarium inoculated leaves. Threonine from two different commercial suppliers was tested with identical outcomes.
Water
THR
Water
THR
Ler0
eds1-2
