# Supplementary material for: Mutations in the Arabidopsis homoserine kinase gene DMR1 confer enhanced resistance to Fusarium culmorum and F. graminearum
Source: BMC Plant Biol. 2014 Nov 29;14:317. doi: 10.1186/s12870-014-0317-0 (PMC4258817; doi:10.1186/s12870-014-0317-0)
Supplement: Additional file 4: Figure S4. — Homoserine treatment reduces F. culmorum growth in point inoculated eds1-2 siliques. Tip-wounded eds1-2 siliques were treated with 10 mM L-homoserine (LHS), D-homoserine (DHS) or sterile water coincident with F. culmorum inoculation. Amino acid/water treatment was repeated for 5 dpi. Images show opened silique sections at 8 dpi. Tissue necrosis and fungal growth is evident in the pericarp (P) and seed (S) of water and D-homoserine (DHS) treated siliques. L-homoserine (LHS) treated siliques have predominantly uninfected pericarps, but some externally uninfected LHS treated siliques revealed, when opened, the presence of fungal colonisation within the silique (far right). [file 12870_2014_317_MOESM4_ESM.pptx]

## Slide 1
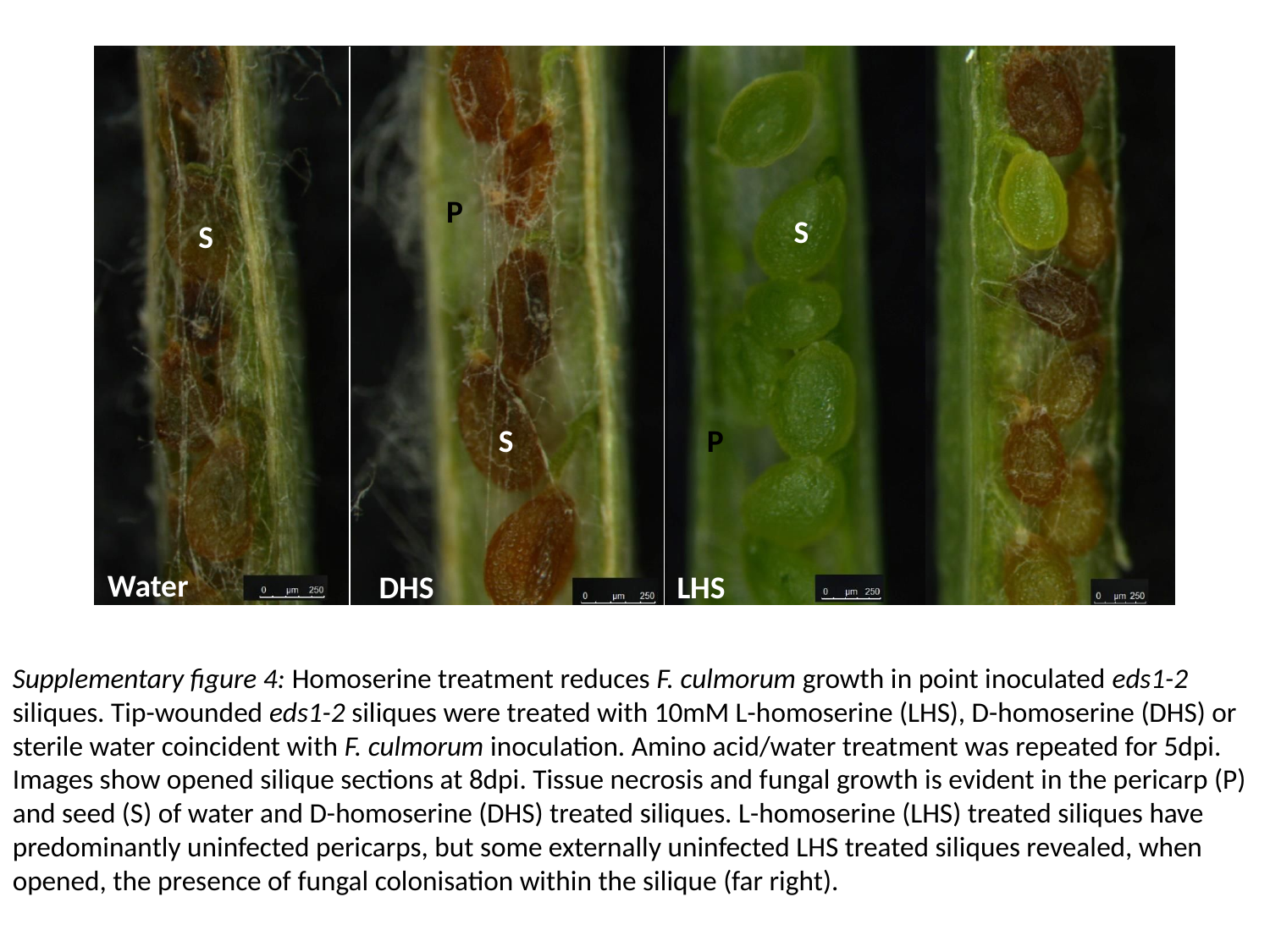

P
S
S
S
P
Water
DHS
LHS
Supplementary figure 4: Homoserine treatment reduces F. culmorum growth in point inoculated eds1-2 siliques. Tip-wounded eds1-2 siliques were treated with 10mM L-homoserine (LHS), D-homoserine (DHS) or sterile water coincident with F. culmorum inoculation. Amino acid/water treatment was repeated for 5dpi. Images show opened silique sections at 8dpi. Tissue necrosis and fungal growth is evident in the pericarp (P) and seed (S) of water and D-homoserine (DHS) treated siliques. L-homoserine (LHS) treated siliques have predominantly uninfected pericarps, but some externally uninfected LHS treated siliques revealed, when opened, the presence of fungal colonisation within the silique (far right).
