# Supplementary material for: Mutations in the Arabidopsis homoserine kinase gene DMR1 confer enhanced resistance to Fusarium culmorum and F. graminearum
Source: BMC Plant Biol. 2014 Nov 29;14:317. doi: 10.1186/s12870-014-0317-0 (PMC4258817; doi:10.1186/s12870-014-0317-0)
Supplement: Additional file 5: Figure S5. — Differences in developmental morphology and senescence between the dmr1-1 and dmr1-2 mutant alleles and eds1-2. (a, b) Rosette diameter is reduced in 5-week old plants of genotype dmr1-2 compared to eds1-2. (c) Leaf number is comparable between genotypes. (d, e) Leaf senescence is delayed in both dmr1-1 and dmr1-2. Panel d shows the appearance of the rosettes of flowering plants at 14 days post flowering. (f) Silique number was equivalent between all genotypes throughout seed set. These phenotypes were observed across multiple experimental replicates. Asterisks indicate significant difference from eds1-2. *p < 0.05, **p = <0.01 (b – ANOVA, e – Regression analysis). [file 12870_2014_317_MOESM5_ESM.pptx]

## Slide 1
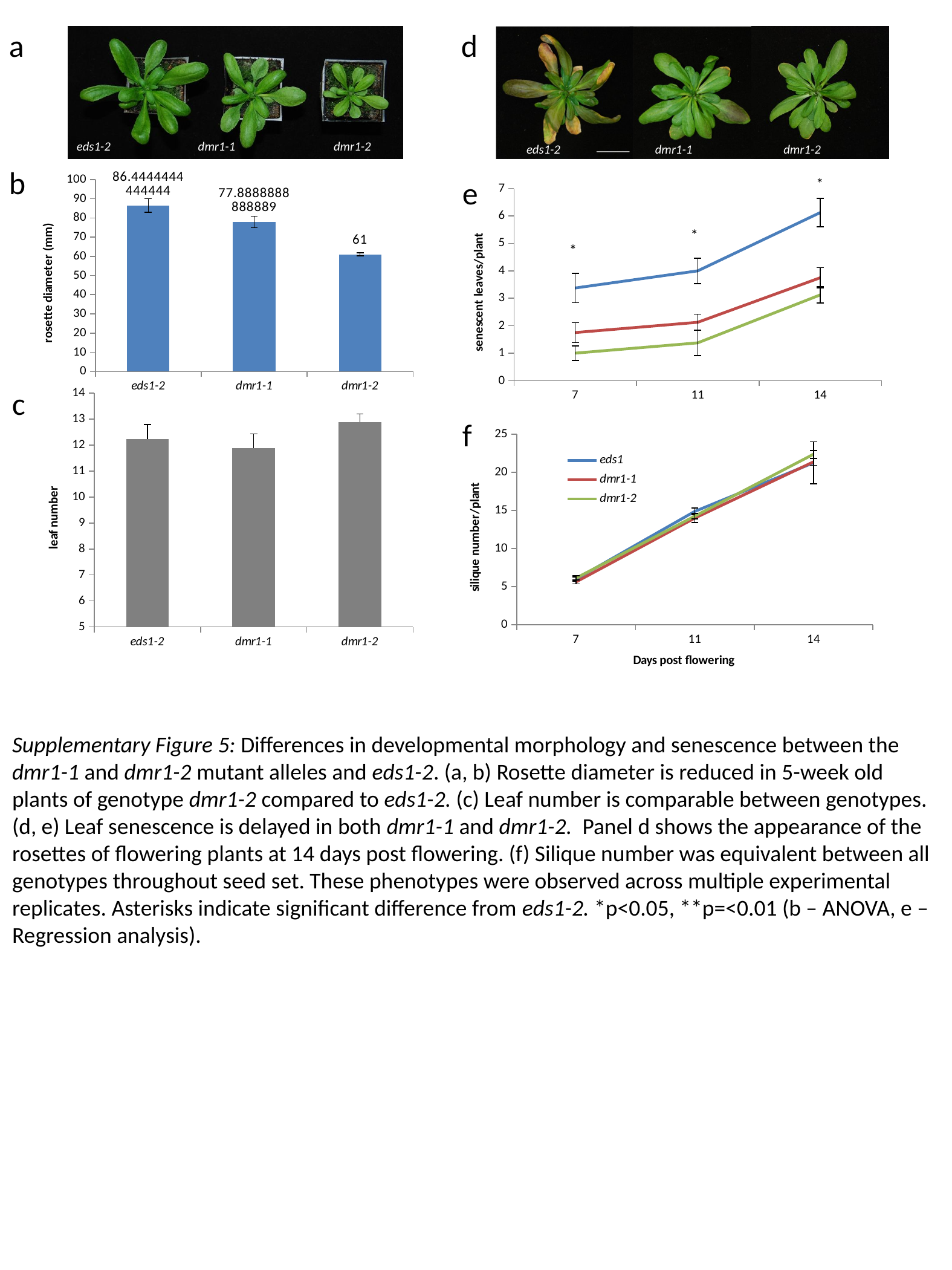

a
d
eds1-2
dmr1-1
dmr1-2
eds1-2
dmr1-1
dmr1-2
b
e
### Chart
| Category | eds1 | dmr1-1 | dmr1-2 |
|---|---|---|---|
| 7 | 3.375 | 1.75 | 1.0 |
| 11 | 4.0 | 2.125 | 1.375 |
| 14 | 6.124999999999999 | 3.75 | 3.125 |c
### Chart
| Category | |
|---|---|
| eds1-2 | 12.222222222222221 |
| dmr1-1 | 11.88888888888889 |
| dmr1-2 | 12.88888888888889 |f
### Chart
| Category | eds1 | dmr1-1 | dmr1-2 |
|---|---|---|---|
| 7 | 6.0 | 5.624999999999999 | 6.124999999999999 |
| 11 | 14.875 | 14.0 | 14.25 |
| 14 | 21.25 | 21.375 | 22.375 |
### Chart
| Category | |
|---|---|
| eds1-2 | 86.44444444444444 |
| dmr1-1 | 77.88888888888889 |
| dmr1-2 | 61.0 |*
*
*
Supplementary Figure 5: Differences in developmental morphology and senescence between the dmr1-1 and dmr1-2 mutant alleles and eds1-2. (a, b) Rosette diameter is reduced in 5-week old plants of genotype dmr1-2 compared to eds1-2. (c) Leaf number is comparable between genotypes. (d, e) Leaf senescence is delayed in both dmr1-1 and dmr1-2. Panel d shows the appearance of the rosettes of flowering plants at 14 days post flowering. (f) Silique number was equivalent between all genotypes throughout seed set. These phenotypes were observed across multiple experimental replicates. Asterisks indicate significant difference from eds1-2. *p<0.05, **p=<0.01 (b – ANOVA, e – Regression analysis).
