# Supplementary material for: Mutations in the Arabidopsis homoserine kinase gene DMR1 confer enhanced resistance to Fusarium culmorum and F. graminearum
Source: BMC Plant Biol. 2014 Nov 29;14:317. doi: 10.1186/s12870-014-0317-0 (PMC4258817; doi:10.1186/s12870-014-0317-0)
Supplement: Additional file 6: Figure S6. — Effect of L-homoserine application on Fusarium infection of wheat. Spikes of wheat cultivar Apogee were point inoculated with F. graminearum and then treated with either L-homoserine (LHS) D-homoserine (DHS) or water for 7 days. The number of bent awns (a) and bleached spikelets (b) along with grain weight (c) and number (d) per plant were assessed at 10 dpi. (*) The number of bleached spikelets was significantly lower in LHS treated plants (p = 0.03). No statistically significant difference between treatments was found for other parameters (ANOVA, p = >0.05). Bar = standard error. [file 12870_2014_317_MOESM6_ESM.pptx]

## Slide 1
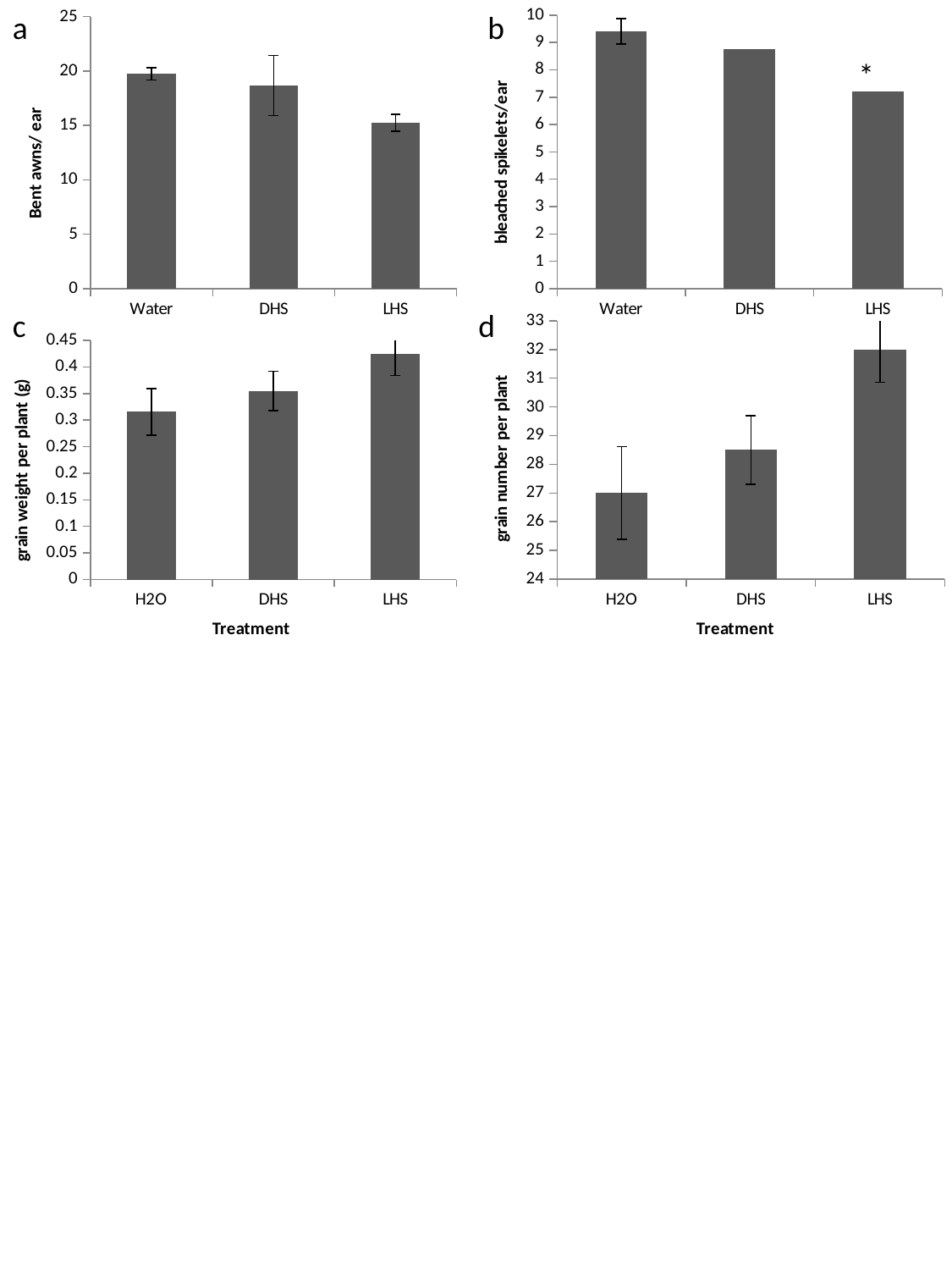

### Chart
| Category | |
|---|---|
| Water | 9.4 |
| DHS | 8.75 |
| LHS | 7.2 |
### Chart
| Category | |
|---|---|
| Water | 19.75 |
| DHS | 18.666666666666668 |
| LHS | 15.25 |a
b
c
d
### Chart
| Category | |
|---|---|
| H2O | 27.0 |
| DHS | 28.5 |
| LHS | 32.0 |
### Chart
| Category | |
|---|---|
| H2O | 0.3154 |
| DHS | 0.35500000000000004 |
| LHS | 0.42400000000000004 |*
