# Supplementary material for: Mutations in the Arabidopsis homoserine kinase gene DMR1 confer enhanced resistance to Fusarium culmorum and F. graminearum
Source: BMC Plant Biol. 2014 Nov 29;14:317. doi: 10.1186/s12870-014-0317-0 (PMC4258817; doi:10.1186/s12870-014-0317-0)
Supplement: Additional file 7: Figure S7. — GENEVESTIGATOR analysis of the expression profile of Arabidopsis DMR1. A) Tissue specific expression levels across different floral tissues. B) Development stage specific expression levels. [file 12870_2014_317_MOESM7_ESM.pptx]

## Slide 1
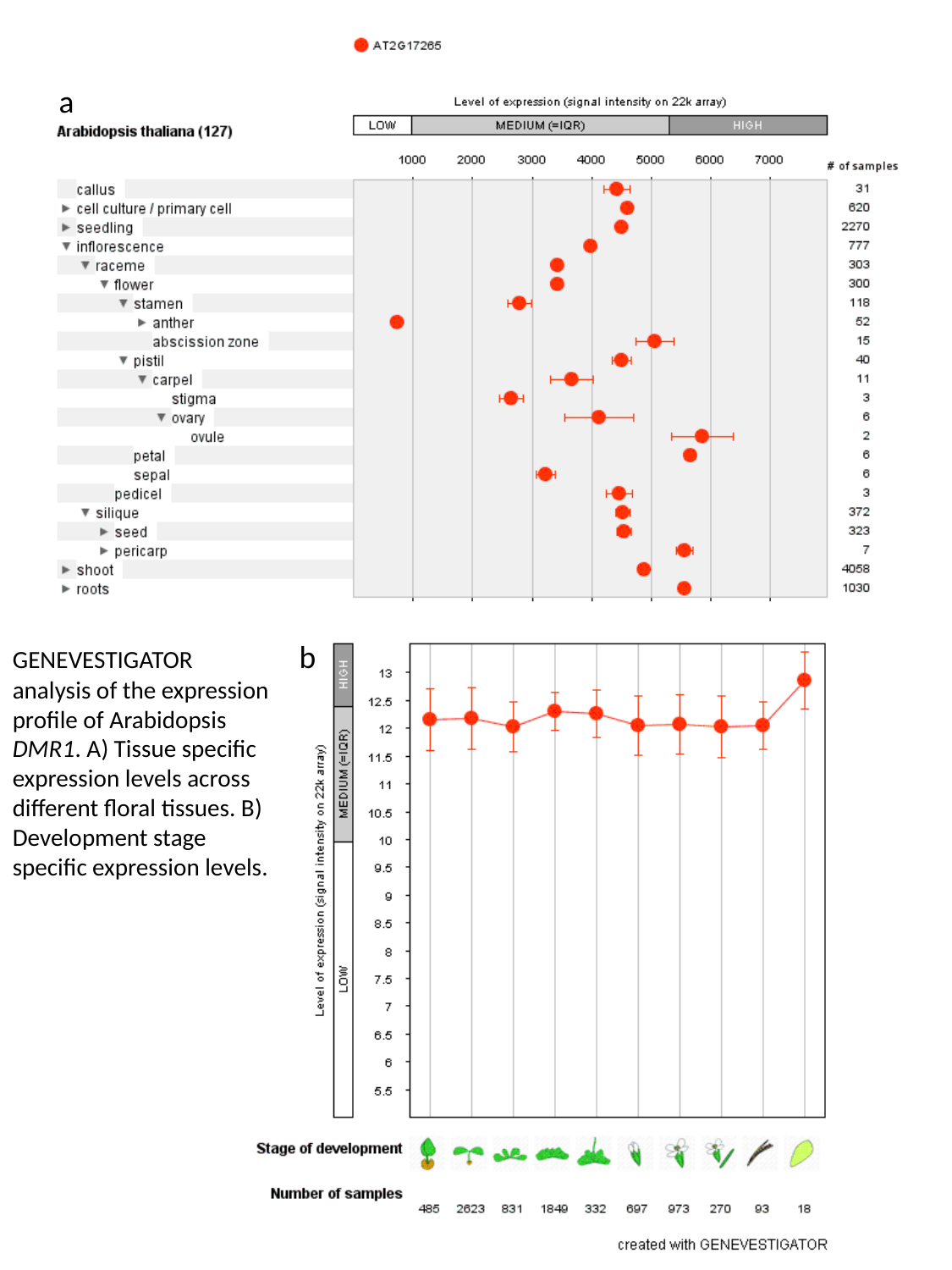

a
b
GENEVESTIGATOR analysis of the expression profile of Arabidopsis DMR1. A) Tissue specific expression levels across different floral tissues. B) Development stage specific expression levels.
