# Supplementary material for: Mutations in the Arabidopsis homoserine kinase gene DMR1 confer enhanced resistance to Fusarium culmorum and F. graminearum
Source: BMC Plant Biol. 2014 Nov 29;14:317. doi: 10.1186/s12870-014-0317-0 (PMC4258817; doi:10.1186/s12870-014-0317-0)
Supplement: Additional file 8: Table S1. — Scoring of Fusarium disease in Arabidopsis floral and silique tissue, adapted from Urban et al. [9]. Plants were given separate scores for floral and silique infection from 0 (no disease) to 7 (constriction of the main stem). The intermediate scores of 2 and 4 (F), and 2, 4 and 6 (S) were reserved for when all the tissue on a single plant exhibited the disease phenotype described for the preceding score. [file 12870_2014_317_MOESM8_ESM.pptx]

## Slide 1
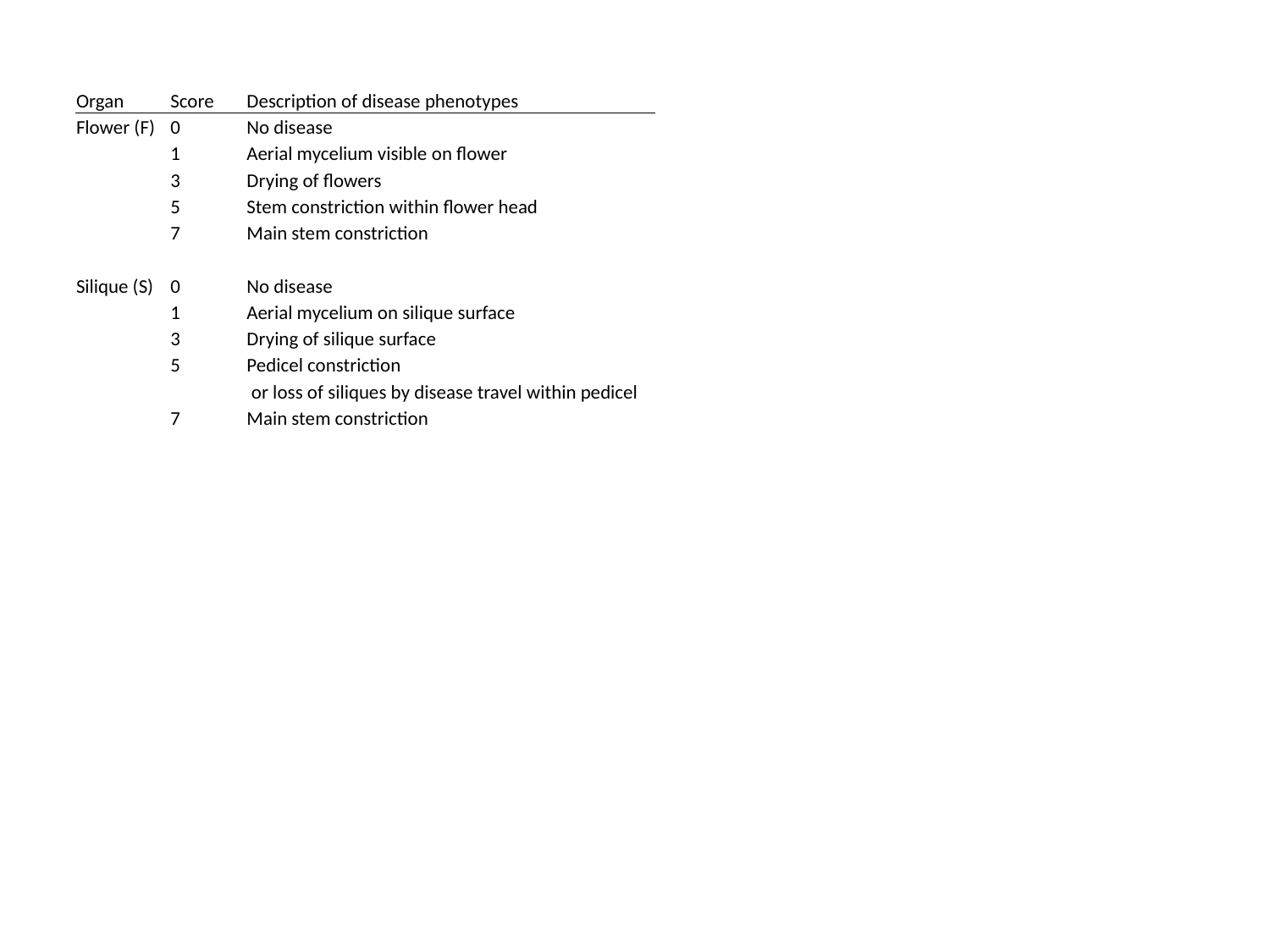

| Organ | Score | Description of disease phenotypes |
| --- | --- | --- |
| Flower (F) | 0 | No disease |
| | 1 | Aerial mycelium visible on flower |
| | 3 | Drying of flowers |
| | 5 | Stem constriction within flower head |
| | 7 | Main stem constriction |
| | | |
| Silique (S) | 0 | No disease |
| | 1 | Aerial mycelium on silique surface |
| | 3 | Drying of silique surface |
| | 5 | Pedicel constriction |
| | | or loss of siliques by disease travel within pedicel |
| | 7 | Main stem constriction |
